# Supplementary material for: Dengue virus preferentially uses human and mosquito non-optimal codons
Source: Mol Syst Biol. 2024 Jul 22;20(10):1085–108. doi: 10.1038/s44320-024-00052-7 (PMC11450187; doi:10.1038/s44320-024-00052-7)
Supplement: Supplementary file 8 — Expanded View Figures [file 44320_2024_52_MOESM8_ESM.pdf]

Expanded View Figures

Figure EV1. Dengue virus serotypes preferentially use nonoptimal codons relative to human.

(**A**) Heatmap showing human CSC. Optimal codons highlighted in red, nonoptimal codons highlighted in blue. Scale bar indicated. (**B**) Scatterplot showing the RSCU fold change (relative to human) for DENV1, DENV3 and DENV4 and human codon stability coefficient (CSC). $R = -0.31$, $P = 0.016$ for DENV1, $R = -0.28$, $P = 0.034$ for DENV3, $R = -0.26$, $P = 0.047$ for DENV4. Spearman rank correlation. (**C**) Scatterplot showing the RSCU fold change (relative to human) for DENV2 3′UTR in the three frames and human codon stability coefficient (CSC). Spearman correlation coefficients and p values indicated, Spearman rank correlation. (**D**) Matrix showing the four DENV serotypes' (DENV1–4) similarity. Lower triangle indicates amino acid similarity, upper triangle indicates nucleotide similarity.

 © The Author(s)

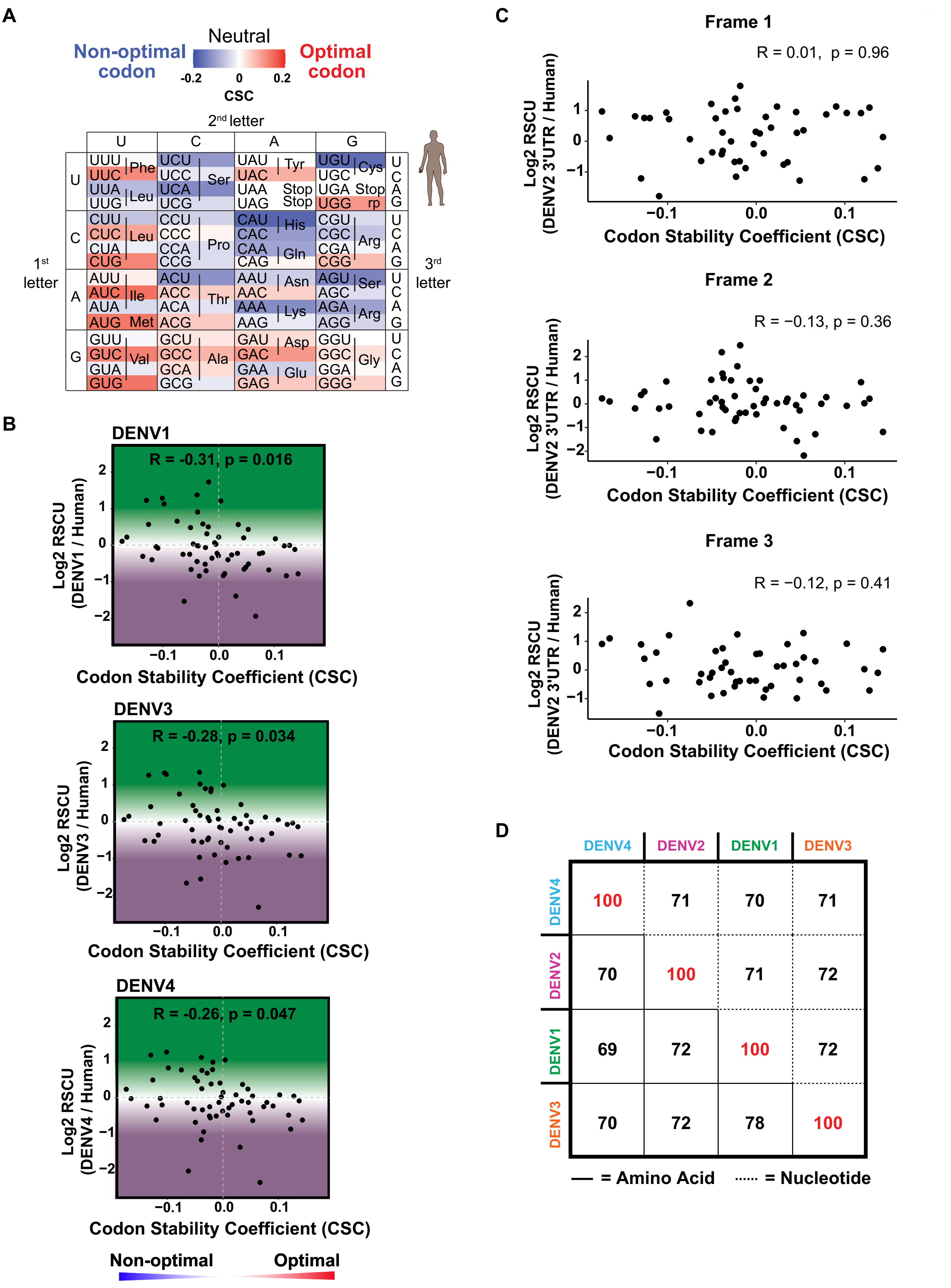
A
Neutral
Non-optimal codon
Optimal codon
-0.2 0 0.2
CSC
2nd letter
U C A G
1st letter
3rd letter
UUU UUC Phe
UUA UUG Leu
UCU UCC UCA UCG Ser
UAU UAC Tyr
UAA Stop
UAG Stop
UGU UGC Cys
UGA Stop
UGG Trp
CUU CUC CUA CUG Leu
CCU CCC CCA CCG Pro
CAU CAC His
CAA CAG Gln
CGU CGC CGA CGG Arg
AUU AUC AUA Ile
AUG Met
ACU ACC ACA ACG Thr
AAU AAC Asn
AAA AAG Lys
AGU AGC Ser
AGA AGG Arg
GUU GUC GUA GUG Val
GCU GCC GCA GCG Ala
GAU GAC Asp
GAA GAG Glu
GGU GGC GGA GGG Gly
B
DENV1
R = -0.31, p = 0.016
Log2 RSCU (DENV1 / Human)
Codon Stability Coefficient (CSC)
DENV3
R = -0.28, p = 0.034
Log2 RSCU (DENV3 / Human)
Codon Stability Coefficient (CSC)
DENV4
R = -0.26, p = 0.047
Log2 RSCU (DENV4 / Human)
Codon Stability Coefficient (CSC)
Non-optimal
Optimal
C
Frame 1
R = 0.01, p = 0.96
Log2 RSCU (DENV2 3'UTR / Human)
Codon Stability Coefficient (CSC)
Frame 2
R = −0.13, p = 0.36
Log2 RSCU (DENV2 3'UTR / Human)
Codon Stability Coefficient (CSC)
Frame 3
R = −0.12, p = 0.41
Log2 RSCU (DENV2 3'UTR / Human)
Codon Stability Coefficient (CSC)
D
DENV4 DENV2 DENV1 DENV3
DENV4 100 71 70 71
DENV2 70 100 71 72
DENV1 69 72 100 72
DENV3 70 72 78 100
— = Amino Acid
······ = Nucleotide

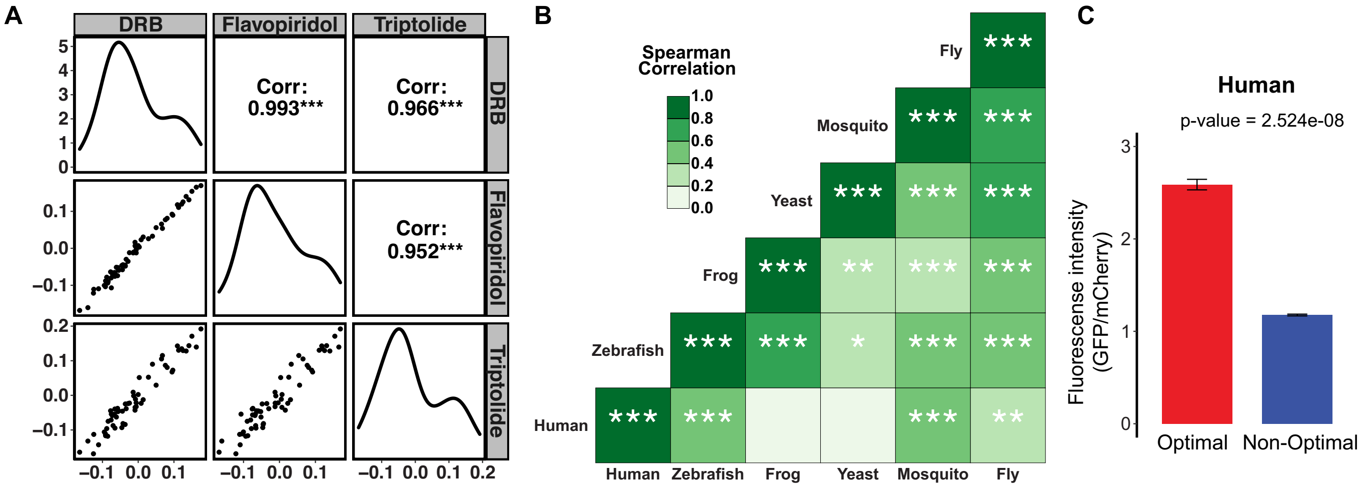

Figure EV2. Codon optimality in mosquito and across species.

(**A**) Pair plot showing the CSC calculated in mosquito C6/36 cells using indicated transcription inhibitors. Lower triangle shows scatterplots of CSCs between inhibitors. Diagonal shows density plot of CSC for each inhibitor. Upper triangle shows Pearson correlation coefficient of CSCs between inhibitors. (**B**) Heatmap showing Spearman rank correlations between known CSCs in indicated species. $P = 8.91e{-}05$ for Human/Mosquito, $P = 1.08e{-}05$ for Human/Zebrafish, $P = 4.13e{-}01$ for Human/Frog, $P = 0.343$ for Human/Yeast, $P = 7.94e{-}03$ for Human/Fly, $P = 3.25e{-}05$ for Mosquito/Zebrafish, $P = 9.29e{-}03$ for Mosquito/Frog, $P = 0.000181$ for Mosquito/Yeast, $P = 4.13e{-}14$ for Mosquito/Fly, $P = 8.70e{-}09$ for Zebrafish/Frog, $P = 0.0295$ for Zebrafish/Yeast, $P = 4.87e{-}06$ for Zebrafish/Fly, $P = 0.00562$ for Frog/Yeast, $P = 9.31e{-}06$ for Frog/Fly, $P = 1.97e{-}07$ for Yeast/Fly. Color of the tile indicates correlation coefficient. $*P < 0.05$, $**P < 0.01$, $***P < 0.001$. (**C**) 1nt frameshift reporters designed based on mosquito codon optimality were co-transfected into 293T human cells with a vector encoding for mCherry as an internal control. Bar plots showing that the 1nt frameshift reporter enriched in optimal codons displayed higher GFP/mCherry fluorescence intensity than its nonoptimal counterpart measured by flow cytometry analysis. Results are shown as the averages of GFP/mCherry fluorescence intensity ± standard error of the mean from two independent experiments with four biological replicates per experiment ($P = 2.524e{-}8$, unpaired t test).

 © The Author(s)

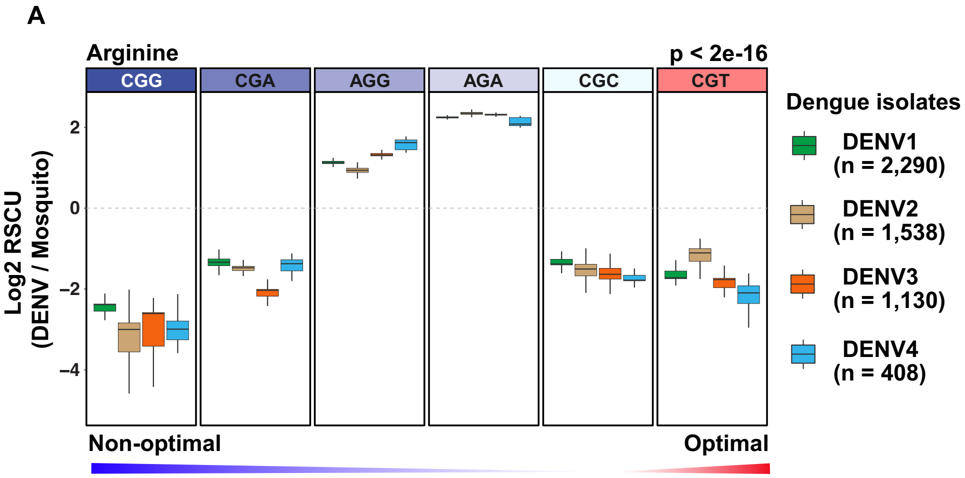

Figure EV3. Arginine codon preference of dengue virus isolates relative to mosquito.

(**A**) Boxplot showing the RSCU fold change (relative to mosquito) of the synonymous codons encoding arginine for 5,366 DENV isolates spanning the four serotypes (DENV1–4). Mosquito CSC indicated by color of codon (red = optimal, blue = nonoptimal). *P* < 2e-16, ANOVA, RSCU fold change relative to mosquito ~ codon. For each boxplot, the median values are depicted as the center (50th percentile). The minima are the smallest data point within 1.5 times the interquartile range below the first quartile (Q1). The maxima are the largest data point within 1.5 times the interquartile range above the third quartile (Q3). The box is defined by the first quartile (Q1–25th percentile) and the third quartile (Q3–75th percentile). The difference between Q3 and Q1 represents the interquartile range. Whiskers extend from the edges of the box to the smallest (minima) and largest (maxima) values within 1.5 times the interquartile range from Q1 and Q3.

© The Author(s)

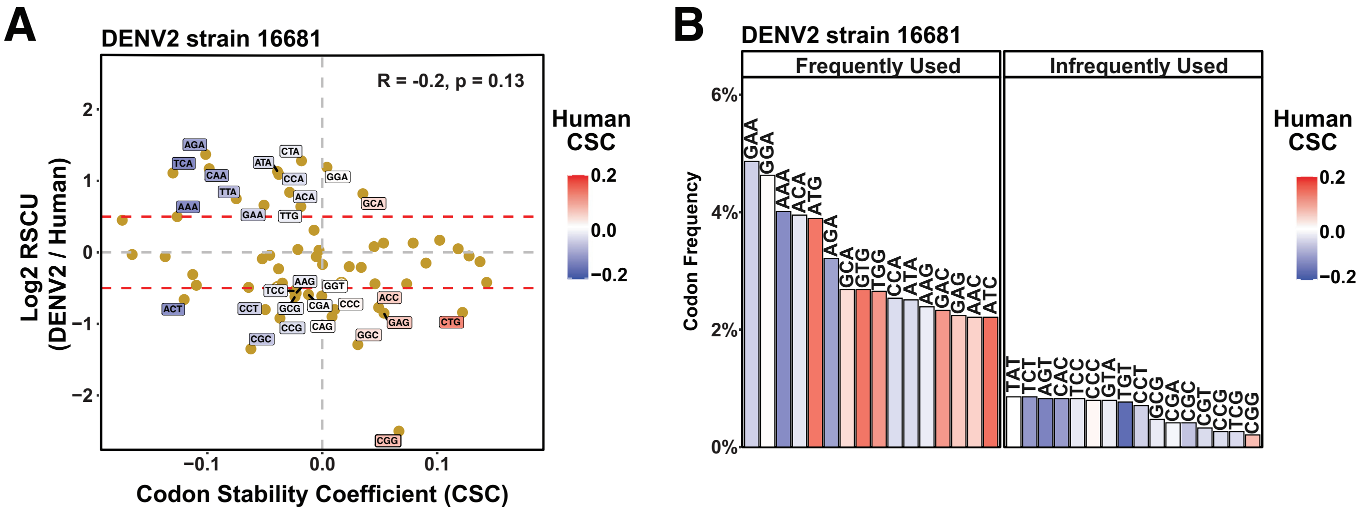

Figure EV4. Classification of dengue virus 2 codons based on preference relative to human and usage.

(**A**) Scatterplot showing the RSCU fold change (relative to human) for DENV2 strain 16681 and human codon stability coefficient (CSC). Labels indicate 'preferentially used' (log2(RSCU fold change relative to human) ≥0.5) and 'not preferentially used' (log2(RSCU fold change relative to human) ≤ −0.5). $R = -0.2$, $P = 0.13$, Spearman rank correlation. (**B**) Barplot showing the frequency of the 16 most used ('frequently used') and the 16 least used ('infrequently used') codons in the DENV2 strain 16681 genome.

 © The Author(s)
